# Prevalence of fundus tessellation and its associated factors in Chinese children and adolescents with high myopia

**DOI:** 10.1111/aos.14826

**Published:** 2021-02-24

**Authors:** Tianyu Cheng, Junjie Deng, Xian Xu, Bo Zhang, Jingjing Wang, Shuyu Xiong, Yuchen Du, Suqin Yu, Wei Gong, Huijuan Zhao, Mengli Luan, Ying Fan, Jianfeng Zhu, Haidong Zou, Xun Xu, Xiangui He

**Affiliations:** ^1^ Shanghai Eye Disease Prevention and Treatment Center Shanghai Eye Hospital Shanghai Children and Adolescent Myopia Prevention and Treatment Technology Center Shanghai China; ^2^ Department of Ophthalmology Shanghai General Hospital Shanghai Jiao Tong University School of Medicine National Clinical Research Center for Eye Diseases Shanghai Key Laboratory of Ocular Fundus Diseases Shanghai Engineering Center for Visual Science and Photomedicine Shanghai Engineering Center for Precise Diagnosis and Treatment of Eye Diseases Shanghai China

**Keywords:** children, choroidal thickness, fundus tessellation, grading, high myopia

## Abstract

**Purpose:**

To investigate the prevalence and associated factors of fundus tessellation in highly myopic children and adolescents.

**Methods:**

A total of 513 high myopes (spherical equivalent [SE] ≤ −5.0 D, 4–19 years of age) without any advanced pathological myopic lesions were enrolled. Fundus photographs and choroidal thickness (ChT) data were collected by SS‐OCT. A novel grading approach was adopted to classify fundus tessellation into four categories on colour fundus photography, referring to the location of tessellation divided by an Early Treatment Diabetic Retinopathy Study grid centred on the fovea, through which closer to the fovea represents higher grades of fundus tessellation. Peripapillary atrophy (PPA) area and ovality index were also measured.

**Results:**

Among the participants, with a mean age of 13.47 ± 3.13 years and mean SE of − 8.34 ± 1.91 D, there were 29 (5.7%), 95 (18.5%), 233 (45.4%) and 156 (30.4%) participants with grade 0 to grade 3 fundus tessellation, respectively. The ChT in both the macular and peripapillary area was negatively correlated with the fundus tessellation grade (*R* = −0.763 and −0.537, respectively, all p < 0.001). Higher grades of fundus tessellation were independently associated with thinner macular ChT (OR = 1.734, 95% CI: 1.621–1.856, p < 0.001), longer axial length (OR = 1.368, 95% CI: 1.105–1.695, p = 0.004), larger PPA area (OR = 1.391, 95% CI: 1.073–1.802, p = 0.013) and the female sex (OR = 1.605, 95% CI: 1.092–2.359, p = 0.016).

**Conclusion:**

The fundus tessellation grade could reflect the ChT, representing the severity of myopic maculopathy among young high myopes who rarely had any advanced lesions of pathological myopia. Fundus tessellation grade might be a potential index for assessing early‐stage myopic maculopathy in children and adolescents.

## Introduction

The prevalence of myopia and high myopia has risen dramatically over the past few decades worldwide, especially in Asian countries, which becomes a major global public health problem (Morgan et al. 2012; Holden et al. 2016; Morgan et al. 2018; Dong et al. 2020). Complications related to high myopia, such as myopic maculopathy, myopic optic neuropathy, glaucoma and retinal detachment, could result in irreversible vision impairment and heavy financial burdens (Iwase et al. 2006; Haarman et al. 2020). Myopic maculopathy has been reported to be the second most common cause of blindness in China and the major reason for blindness in Shanghai (Xu et al. 2006; Wu et al. 2011).

Fundus tessellation, the early stage of myopic maculopathy, is described as well‐defined choroidal vessels that can be observed clearly around the fovea as well as around the arcade vessels according to the International Photographic Classification and Grading System (Ohno‐Matsui et al. 2015). It has been reported to be a relatively stable stage during the progression of myopic maculopathy and the most common myopic lesion in children and adolescents with high myopia (Ohno‐Matsui et al. 2015).

However, choroidal thinness could also exist in eyes without subfoveal tessellation in children and adolescents (Guo et al. 2019). As the current grading system is based on data from the adult population, the strict criteria for fundus tessellation may lead to the omission of young patients. In addition, approximately 10% of eyes with fundus tessellation could show progression to diffuse chorioretinal atrophy (DCA), and pathological lesions, such as lacquer cracks and choroidal neovascularization (CNV), may also develop in eyes with fundus tessellation only (Hayashi et al. 2010). Several studies have previously reported the factors associated with fundus tessellation or myopic maculopathy in adults (Yan et al. 2015; Fang et al. 2018; Xiao et al. 2018; Chen et al. 2019), but few studies have investigated these factors in highly myopic children and adolescents who were at a higher potential risk of progression, considering that they were still undergoing rapid ocular development. Therefore, a more detailed diagnostic grading system for fundus tessellation is needed to investigate the associated factors and monitor those with a high risk of progression.

He et al. recently proposed a novel grading system for DCA that incorporates the Early Treatment Diabetic Retinopathy Study (ETDRS) grid for fundus photographs and found that the choroidal thickness (ChT) decreased with increasing DCA level (Liu et al. 2020). Additionally, based on the knowledge that the ChT increases from perifoveal regions to parafoveal and subfoveal regions in healthy children and adolescents (Deng et al. 2018), fundus tessellation, which represents an area with a thinner ChT, may develop from the perimeter zone to the fovea with the progression of myopia. Thus, He’s grading method for DCA could also be used in grading fundus tessellation. In this study, we aimed to investigate the prevalence of fundus tessellation in a young Chinese population with high myopia by applying this new grading method and explore the associated factors.

## Methods

### Participants

This was a cross‐sectional study based on the established refractive developmental archives in the Shanghai child and adolescent large‐scale eye (SCALE) study (He et al. 2018). Children and adolescents with high myopia (SE ≤ −5 D, noncycloplegic) were invited for further examinations. A total of 585 subjects were enrolled in this study. Among them, 41 were excluded because of other organic eye diseases (amblyopia, strabismus, congenital cataract and glaucoma), noncooperation with some of the ophthalmic examinations and an unqualified SE after cycloplegia. Additionally, 31 individuals who were classified as having advanced myopic maculopathy (peripapillary or macular DCA) were also excluded. Finally, 513 highly myopic participants aged 4 to 19 years (mean age, 13.47 ± 3.13 years) were included in the final analysis. All the participants and their guardians were informed of the study purpose and protocol. Written informed consent was obtained from guardians and participants older than 12 years, while oral consent was obtained from children younger than 12 years. This study, which was approved by the Shanghai General Hospital Ethics Committee, adhered to the tenets of the Declaration of Helsinki.

### Examinations

All of the participants underwent comprehensive examinations. First, height and weight measurements were conducted. Visual acuity was tested at a 4 m distance using a retro‐illuminated ETDRS chart, including both the uncorrected visual acuity (UCVA) and corrected visual acuity (CVA). The axial length (AL), anterior chamber depth (ACD), central corneal thickness (CCT) and lens thickness (LT) were measured with an IOL master 700 (Carl Zeiss Meditec, Germany), and the intraocular pressure (IOP) was measured with a noncontact tonometer (NT‐510, Nidek, Japan). After a slit lamp examination, which was administered by an ophthalmologist to guarantee safety for cycloplegia, participants received one drop of 0.5% proparacaine (Alcaine, Alcon) in each eye, followed by two drops of 1% cyclopentolate (Cyclogyl, Alcon), with each drop administered 5 min apart. During the cycloplegia procedure, a questionnaire for the participants and their guardians was conducted in question–answer format by a co‐ordinator. The questionnaire included questions about their basic information, myopia onset age and family history of myopia. Approximately 30 min after the last drop of cyclopentolate was administered, the absence of light reflexes and a pupil diameter >6 mm were considered to indicate successful cycloplegia. Then, refraction and corneal curvature measurements were acquired by an autorefractor (KR‐8900, Topcon, Japan). Subjective refraction measurements were also obtained to assess the best‐corrected acuity (BCVA). Finally, a swept‐source optical coherence tomography (SS‐OCT, DRI OCT Triton, Topcon) examination was performed by an experienced examiner from 10:00 AM to 3:00 PM each day to minimize the influence of diurnal variation (Tan et al. 2012). At the same time, 45‐degree colour fundus photographs for both the macular and peripapillary areas were also acquired using the same SS‐OCT system with an inserted digital retinal camera.

### ChT Measurements

The SS‐OCT examination was conducted in a 12‐line radial scan pattern centred on both the fovea and optic disc with a resolution of 1024 for each line. Before scanning, some ocular parameters, such as the spherical power (dioptre, D), cylindrical power (D), AL (mm) and corneal curvature radius (CR, mm), were input to the OCT system to perform calibration for the magnification error, which was primarily associated with the AL. Segmentation of the different layers on the OCT images was automatically completed by built‐in software, followed by manual inspection and correction for misjudgement of the layer borders by an OCT technician (Z.B.). Images with poor quality (signal strength less than 60) were excluded from the final analysis.

The ChT was defined as the vertical distance between Bruch’s membrane and the choroidal‐scleral interface. The ETDRS grid (6 × 6 mm) was centred on both the fovea and optic disc, separating these two regions into nine sectors by three concentric circles: an inner circle (diameter of 1 mm), a middle circle (diameter of 3 mm) and an outer circle (diameter of 6 mm). The inner ring (parafoveal region in the macular area), which was between the inner and middle circles, and the outer ring (perifoveal region in the macular area), which was between the middle and outer circles, were further divided into four quadrants: temporal, superior, nasal and inferior. The average ChT in each sector of the grid was automatically calculated by the built‐in SS‐OCT software. In the macular area, the ChT in all nine sectors was included in the analysis, while in the peripapillary area, only the four sectors in the outer ring were used. The absence of choroidal tissue and irregular topographic maps in the inner ring and centre of the optic disc resulted in unreliable ChT measurements.

### Peripapillary atrophy area, ovality index and torsion measurements

**Fig. 1:**
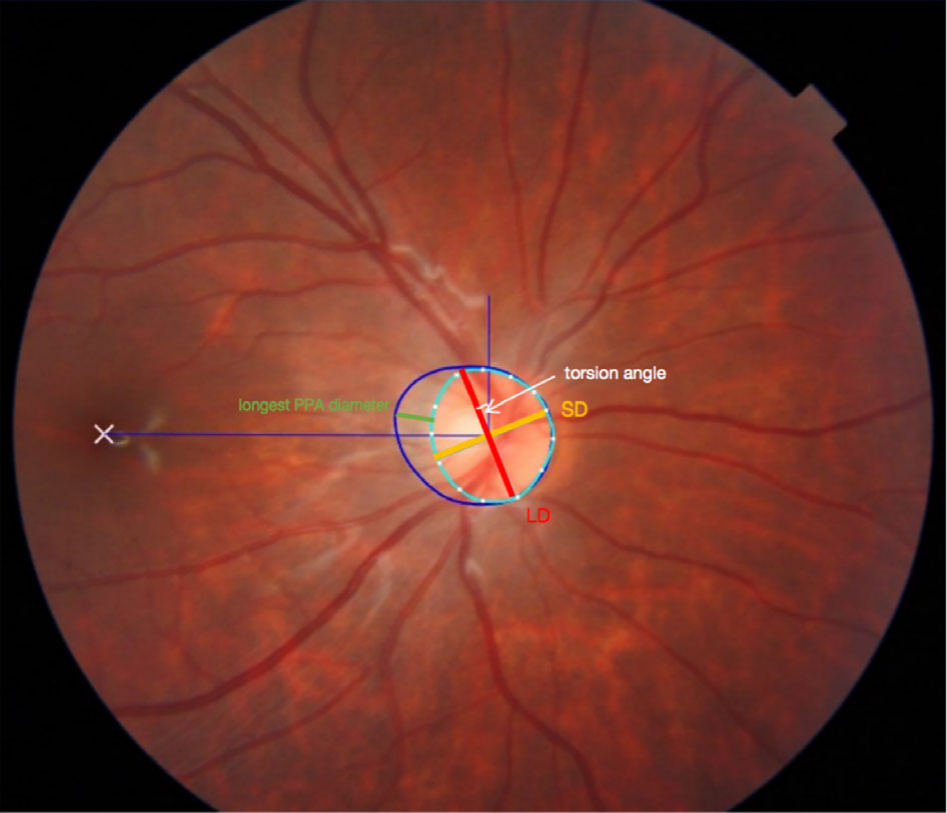
Measurement of peripapillary atrophy (PPA) area, ovality index and torsion angle by in‐house software. The red and orange lines represent the longest diameter (LD) and shortest diameter (SD) of the optic disc, respectively. The PPA area was outlined manually (area between the dark blue line and light blue line), and the area in the pixel was calculated automatically, as well as its longest diameter (the green line). The torsion angle (the white angle) was measured between LD and the line that was perpendicular to the fovea‐disc connecting line.

Colour fundus photographs centred on the optic disc were used for measurement of the peripapillary atrophy (PPA) area, ovality index and optic disc torsion angle. An in‐house annotation software (Fig. 1) was applied to smooth the contours of the delineated regions with B‐spline interpolation and avoid burrs generated by manual errors (De Boor 1978). All measurements were conducted by an experienced ophthalmologist (T.Y.) without any knowledge of participants’ ocular data or diagnosis.

**Fig. 2:**
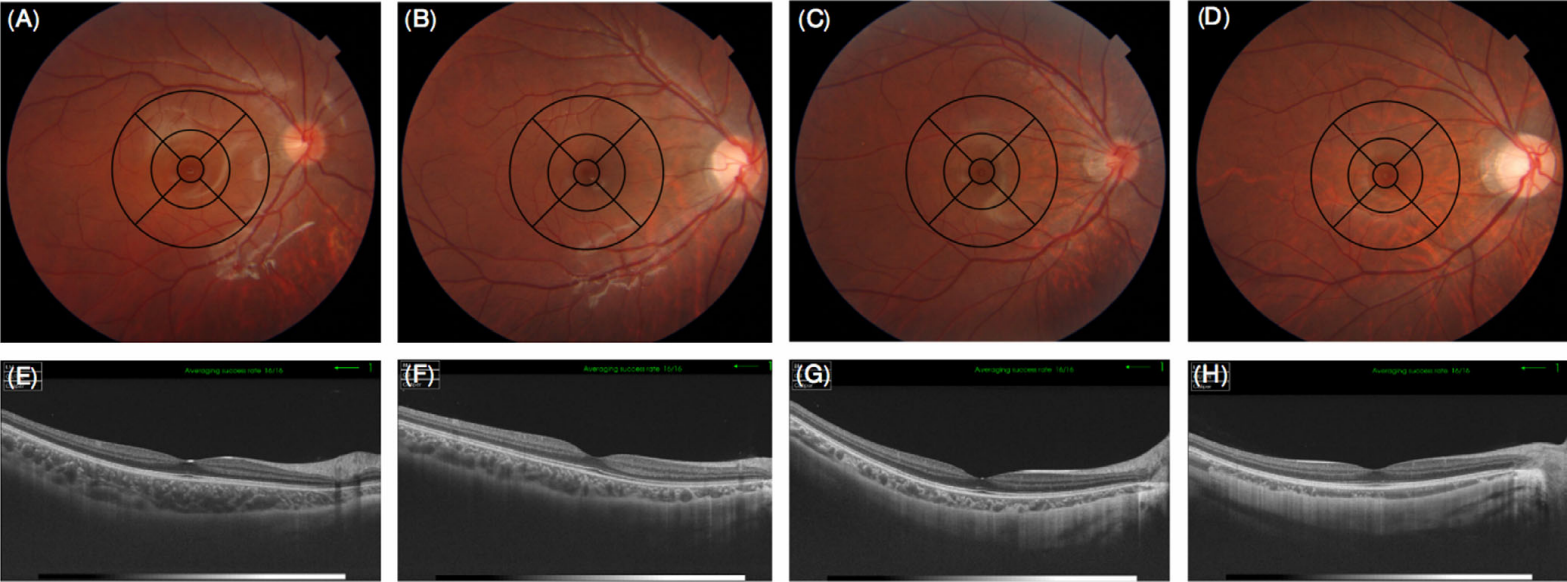
Application of EDTRS grid in fundus tessellation grading. (A–D) Sample graphs for grade 0 (no involvement of the outer circle), grade 1 (involvement of the outer circle), grade 2 (involvement of the middle circle) and grade 3 (involvement of the inner circle), respectively. (E–H) show that the corresponding choroidal thickness had a decreasing trend with fundus tessellation progression.

The PPA area was measured as the total number of pixels using our in‐house annotation software. Then, it was converted from pixels to square millimetres. The magnification was adjusted by the AL based on Littmann’s formula (Bennett et al. 1994; Hirasawa et al. 2014). Optic disc tilt was distinguished by the ovality index, which was defined as the ratio of the longest to the shortest diameter of the optic disc. An optic disc was classified as tilted when the ovality index was ≥1.25 (Vongphanit et al. 2002; Tay et al. 2005; Hosseini et al. 2013). The torsion angle was defined as the angle between the long axis of the optic disc and the vertical line, which was perpendicular to the fovea‐disc connecting line. Inferior torsion (counterclockwise) and superior torsion (clockwise) are presented as positive and negative values, respectively (Park et al. 2012; Lee et al. 2014).

### Grading of fundus tessellation

Fundus tessellation was graded using colour fundus photographs centred on the fovea obtained during the same SS‐OCT examination (Liu et al. 2020). Adopting the ETDRS grid, fundus tessellation was classified into four grades (Fig. 2): no involvement of the outer circle (grade 0, including eyes without tessellation); involvement of the outer circle (grade 1); involvement of the middle circle (grade 2); and involvement of the inner circle (grade 3). When assessing the grade, the contrast, brightness, background pigmentation and quality of the images were all taken into account. Two trained ophthalmologists (T.Y. and J.J.) independently read these photographs in a masked manner to determine the tessellation grade. A senior ophthalmologist (X.X.) was required to make the decision if there was any disagreement regarding the grade. Intraobserver variability was tested by an ophthalmologist (T.Y.) who randomly chose 50 of these photographs and read them twice at an interval of two weeks (kappa = 0.93). Interobserver variability was tested by two ophthalmologists (T.Y. and J.J.) with another 50 randomly chosen photographs (kappa = 0.91).

### Statistical analysis

Statistical analyses were performed with spss (IBM SPSS Statistics, Inc., version 25.0, Chicago, IL, USA). Only data from the right eyes were randomly chosen and included in the final analyses. Continuous variables are described as the mean ± standard deviation, while discrete variables are described as counts (proportions). High myopia was defined as SE ≤−5 D after cycloplegia, and SE was calculated as spherical power + 0.5*cylindrical power. Body mass index was calculated as weight/height^2^ (kg/m^2^). Patients were separated into three groups by age (≤11 years, 11 < age≤14 years, >14 years), AL (≤26 mm, 26 < AL≤27 mm, >27 mm) and SE (≤−9 D, −9 < SE≤−7 D, >−7 D) to explore differences in the tessellation grade distribution. There were four main parts of our analysis. First, we compared the proportions of different tessellation grades according to age, AL and SE by the chi‐square test. Second, we analysed differences in the ChT among tessellation grades in each macular and peripapillary area using analysis of variance (anova). The test for linear trend was performed with a polynominal contrast procedure. The Student–Newman–Keuls (SNK) method was applied for post hoc analysis. Third, a comparison of optic disc morphological parameters and other ocular variables among tessellation grades was also performed. Finally, a multivariate analysis for factors associated with the tessellation grade was conducted with ordinal regression. Statistical significance was defined as p < 0.05 (two‐tailed).

## Results

### General characteristics

A total of 513 fundus images of the right eye were included in the final analysis. The mean age was 13.47 ± 3.13 years (range, 4–19), and 240 (46.8%) of the patients were male. The mean AL was 26.63 ± 1.04 mm (range, 23.36–29.51 mm); the mean SE was − 8.34 ± 1.91 D (range, −14.375 D to − 5.000 D); the mean corneal CR was 7.74 ± 0.26 mm (range, 6.89–8.58 mm); and the mean axial length/corneal curvature ratio (AL/CR) was 3.44 ± 0.11 (range, 3.10–3.75). Fundus tessellation in the macular area was found in 484 (94.3%) cases, with 95 (18.5%), 233 (45.4%) and 156 (30.4%) cases of grade 1, 2 and 3 tessellation, respectively.

### Comparison of age, AL and refraction

**Fig. 3:**
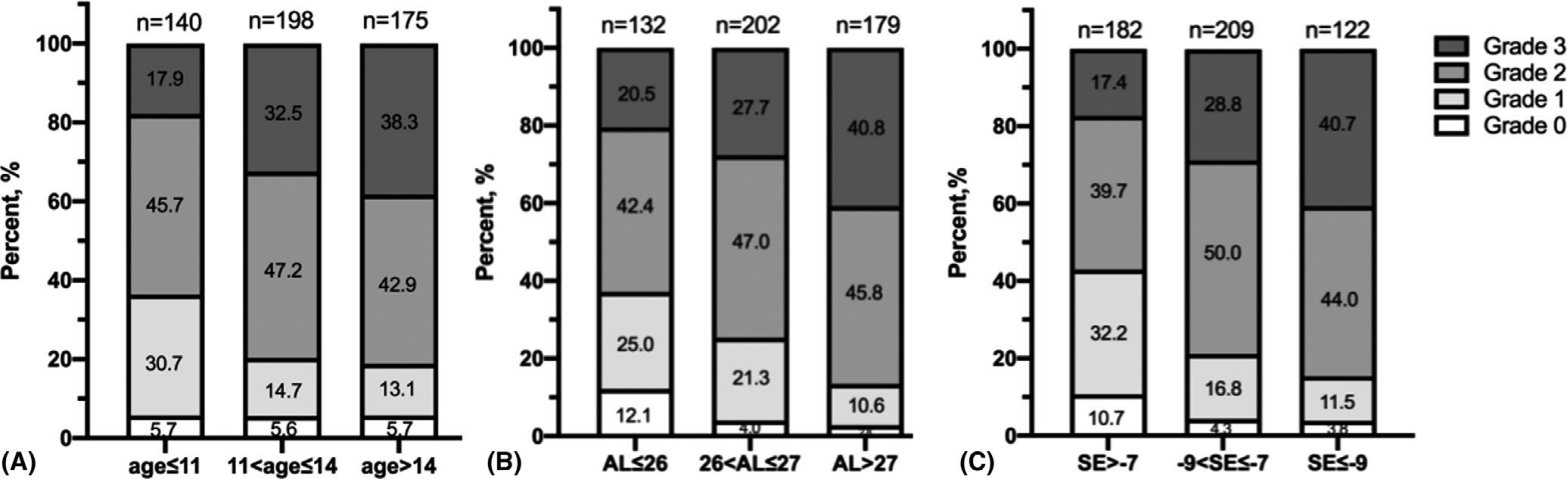
Prevalence of different fundus tessellation grades among age (A), axial length (B) and spherical equivalent (C) groups.

As shown in Fig. 3, a higher degree of fundus tessellation was more common in participants with a more advanced age, a longer AL and more myopic refraction. The proportion of grade 3 macular tessellation was 17.9%, 32.5% and 38.3% in participants aged ≤11 years, >11 and ≤14 years, and > 14 years, respectively (p < 0.001). In eyes with an AL < 26 mm, the proportion of grade 3 macular tessellation (20.5%) was significantly less than that in eyes with a longer AL (26 < AL≤27 mm group, 27.7%; AL > 27 mm group, 40.8%; all p < 0.001). Consistently, the proportion of grade 3 macular tessellation gradually increased as the SE became more myopic (SE>−7 D group, 17.4%; −9 D < SE≤−7 D group, 28.8%; SE≤−9 D group, 40.7%; p < 0.001).

As fundus tessellation progressed, the AL became longer and the SE became more myopic (p < 0.001, p‐trend < 0.001, Table 1). Similarly, the corneal CR became flatter (p = 0.047, p‐trend = 0.031), and AL/CR increased (p < 0.001, p‐trend < 0.001) as the tessellation level increased. However, no significant difference was observed in the AL, SE and AL/CR between the grade 0 and grade 1 groups. Some parameters, such as height and BMI, revealed significant differences among tessellation grades but showed no obvious trend as the tessellation level increased (p < 0.05, p‐trend > 0.05). However, other parameters, such as sex, BCVA, ACD and parental myopia, showed no significant differences among the tessellation grades (all p > 0.05, p‐trend > 0.05).

**Table 1:** Comparison of demographic and ocular parameters among different fundus tessellation grades.

Variables	Grade 0 (*N* = 29)	Grade 1 (*N* = 95)	Grade 2 (*N* = 233)	Grade 3 (*N* = 156)	Total (*N* = 513)	p*	p‐Trend^†^	Post Hoc Test^‡^
Age, year	13.58 ± 2.96	12.38 ± 3.21	13.37 ± 3.09	14.27 ± 2.99	13.47 ± 3.13	<0.001	0.111	Grade1 < Grade0/Grade3
Gender, boy (%)	13(44.8)	52(54.7)	111(47.6)	64(41.0)	240(46.8)	0.203	/	/
Height, cm	159.93 ± 14.49	154.15 ± 16.92	159.00 ± 16.49	161.75 ± 15.21	158.99 ± 16.25	0.004	0.301	Grade1 < Grade3
BMI, kg/m2	22.19 ± 5.07	19.26 ± 3.95	20.14 ± 4.19	20.69 ± 5.47	20.26 ± 4.66	0.012	0.208	Grade0 > Grade1/Grade2
Myopia Onset, year	7.00 ± 2.56	6.51 ± 2.45	6.83 ± 2.42	7.42 ± 2.65	6.95 ± 2.52	0.087	0.363	/
Parental Myopia, *N* (%)								
Both	18(62.1)	46(48.4)	110(47.2)	71(45.5)	245(47.8)	0.382	/	/
Either	10(34.5)	42(44.2)	102(43.8)	78(50.0)	232(45.2)			
Neither	1(3.4)	7(7.4)	21(9.0)	7(4.5)	36(7.0)			
Parental High Myopia, *N* (%)								
Both	4(13.8)	16(16.8)	37(15.9)	21(13.5)	78(15.2)	0.838	/	/
Either	16(55.2)	41(43.2)	106(45.5)	80(51.3)	243(47.4)			
Neither	9(31.0)	38(40.0)	90(38.6)	55(35.3)	192(37.4)			
Axial Length, mm	26.04 ± 1.01	26.27 ± 0.92	26.60 ± 1.01	26.99 ± 1.04	26.63 ± 1.04	<0.001	<0.001	Grade0/Grade1 < Grade2<Grade3
Spherical Equivalent, D	−7.55 ± 2.32	−7.55 ± 1.90	−8.29 ± 1.71	−8.99 ± 2.02	−8.34 ± 1.91	<0.001	<0.001	Grade0/Grade1 > Grade2>Grade3
Intraocular Pressure, mmHg	16.46 ± 3.12	15.56 ± 2.82	15.45 ± 2.81	15.60 ± 2.35	15.57 ± 2.70	0.322	0.114	/
Central Corneal Thickness,	547.62 ± 34.43	544.60 ± 29.50	540.74 ± 32.89	544.70 ± 35.24	543.05 ± 33.10	0.517	0.538	/
Anterior Chamber Depth, mm	3.50 ± 0.34	3.44 ± 0.32	3.46 ± 0.36	3.40 ± 0.33	3.44 ± 0.34	0.322	0.217	/
Lens Thickness, mm	3.43 ± 0.16	3.38 ± 0.15	3.41 ± 0.16	3.41 ± 0.17	3.40 ± 0.16	0.354	0.689	/
Corneal Curvature Radius, mm	7.67 ± 0.27	7.71 ± 0.23	7.72 ± 0.26	7.78 ± 0.26	7.74 ± 0.26	0.047	0.031	Grade0 < Grade3
AL/CR	3.40 ± 0.15	3.41 ± 0.12	3.45 ± 0.10	3.47 ± 0.11	3.44 ± 0.11	<0.001	<0.001	Grade0/Grade1 < Grade2/Grade3
BCVA, logMAR	0.044 ± 0.010	0.028 ± 0.105	0.036 ± 0.092	0.029 ± 0.085	0.033 ± 0.093	0.732	0.512	/

AL/CR = axial length/corneal curvature ratio; BCVA = best‐corrected visual acuity; BMI = body mass index.

* Significant difference was tested by one‐way ANOVA and chi‐square test.

^†^ p‐trend was acquired by trend test.

^‡^ Post hoc tests among tessellation groups were conducted with SNK method.

### Comparison of the ChT

**Fig. 4:**
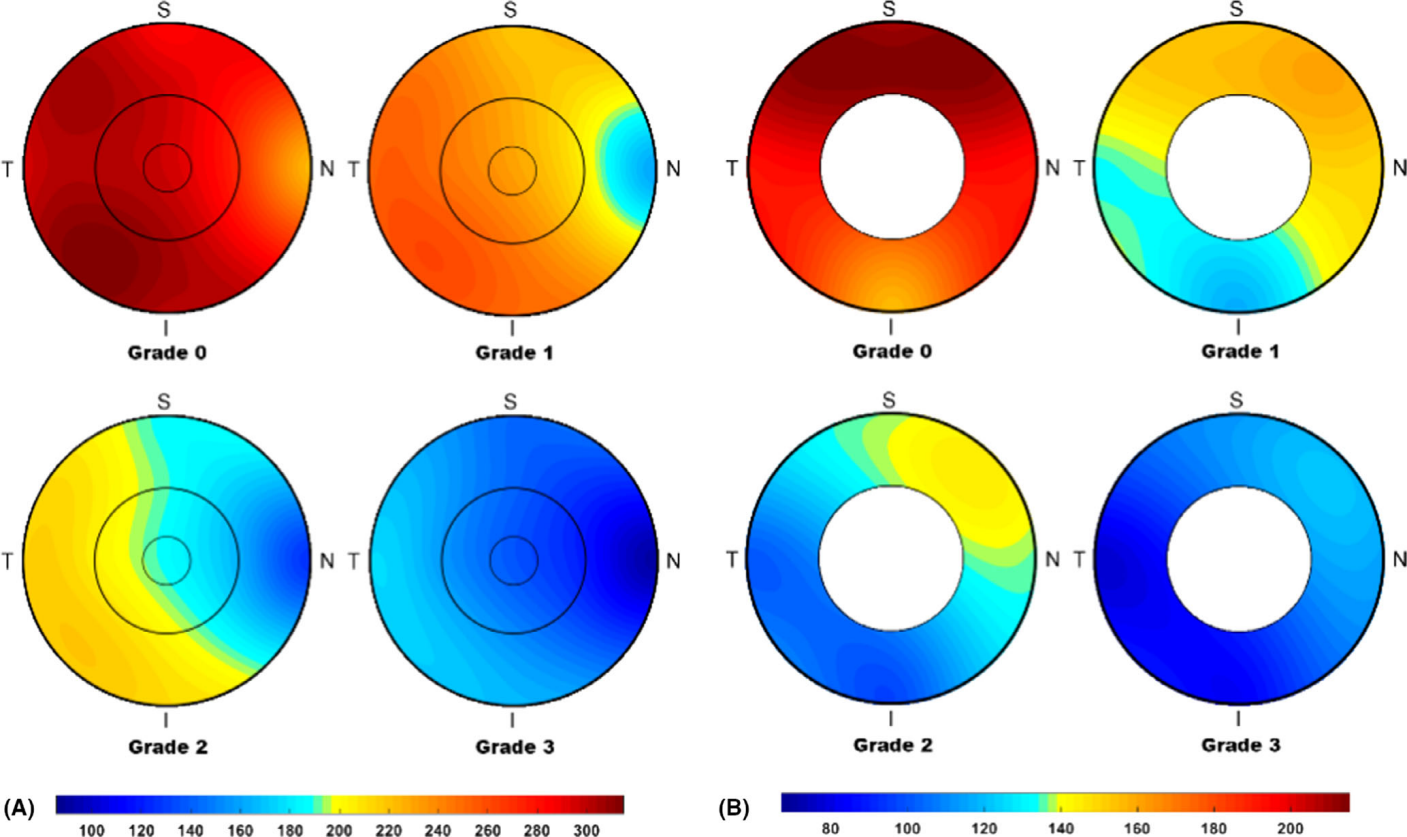
Choroidal thickness in macular (A) and peripapillary areas (B) with increases in fundus tessellation grade.

**Fig. 5:**
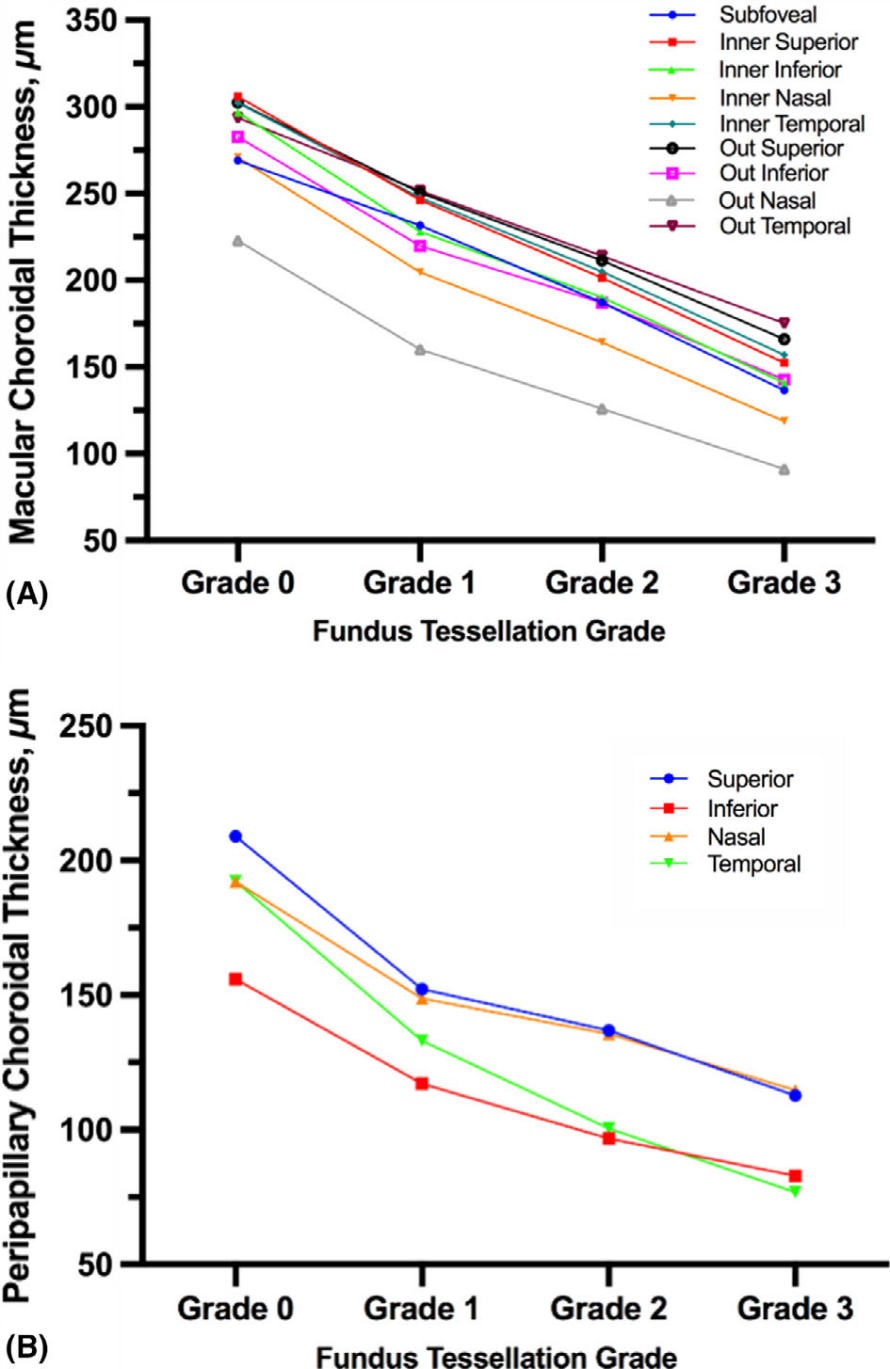
Differences in mean choroidal thickness with increased grades of fundus tessellation in the macular and peripapillary areas.

The ChT in the macular and peripapillary areas for the different tessellation grades is shown in Table 2 and Fig. 4. The mean ChT in the macular area decreased from 280 ± 43 µm in grade 0 fundus tessellation to 223 ± 30 µm in grade 1, to 186 ± 30 µm in grade 2 and to 143 ± 25 µm in grade 3 (p < 0.001, p‐trend < 0.001). All sections in the macular and peripapillary areas showed similar decreasing trends, with the tessellation grade becoming more severe (all p < 0.001, p‐trend < 0.001). The ChT in the nasal region in the macular area and inferotemporal region in the peripapillary area was significantly thinner than that in the other regions (Fig. 4). In the macular area, the change in ChT from grade 0 to 1 in the subfoveal and outer temporal regions was smaller than that in the other regions, while all of the regions in the macular area showed similar changes in ChT beyond grade 1 fundus tessellation (Fig. 5A). In the peripapillary area, the temporal region showed the greatest change in ChT from grade 0 to 3 fundus tessellation (Fig. 5B).

**Table 2:** Comparison of choroidal thickness among different grades of fundus tessellation (Mean ± SD).

Choroidal thickness, µm	Grade 0 (*N* = 29)	Grade 1 (*N* = 95)	Grade 2 (*N* = 233)	Grade 3 (*N* = 156)	Total (*N* = 513)	p*	p‐Trend^†^, ^‡^
Macula	280 ± 43	223 ± 30	186 ± 30	143 ± 25	185 ± 47	<0.001	<0.001
Subfoveal	296 ± 58	232 ± 38	187 ± 35	137 ± 28	186 ± 56	<0.001	<0.001
Inner Superior	306 ± 52	246 ± 40	201 ± 36	152 ± 30	201 ± 55	<0.001	<0.001
Inner Inferior	297 ± 54	228 ± 35	190 ± 35	141 ± 28	188 ± 53	<0.001	<0.001
Inner Nasal	271 ± 55	205 ± 33	164 ± 34	119 ± 28	164 ± 52	<0.001	<0.001
Inner Temporal	302 ± 58	248 ± 41	205 ± 37	157 ± 30	204 ± 54	<0.001	<0.001
Out Superior	302 ± 42	250 ± 41	211 ± 35	166 ± 35	210 ± 52	<0.001	<0.001
Out Inferior	283 ± 45	220 ± 37	187 ± 36	143 ± 29	185 ± 50	<0.001	<0.001
Out Nasal	223 ± 42	160 ± 30	126 ± 32	91 ± 24	127 ± 45	<0.001	<0.001
Out Temporal	294 ± 56	252 ± 37	214 ± 36	175 ± 32	215 ± 49	<0.001	<0.001
Peripapillary	187 ± 31	138 ± 31	117 ± 29	97 ± 25	119 ± 36	<0.001	<0.001
Peripapillary Superior	209 ± 40	152 ± 37	137 ± 37	113 ± 34	137 ± 43	<0.001	<0.001
Peripapillary Inferior	156 ± 32	117 ± 32	97 ± 26	83 ± 22	100 ± 32	<0.001	<0.001
Peripapillary Nasal	192 ± 38	149 ± 39	136 ± 35	115 ± 35	135 ± 40	<0.001	<0.001
Peripapillary Temporal	192 ± 40	133 ± 33	101 ± 32	77 ± 26	105 ± 42	<0.001	<0.001

* Significant difference was tested by one‐way ANOVA.

^†^ p‐trend was acquired by trend test.

^‡^ Post hoc test by SNK method: Grade0 > Grade1>Grade2 > Grade3.

### Comparison of optic disc morphological parameters

**Fig. 6:**
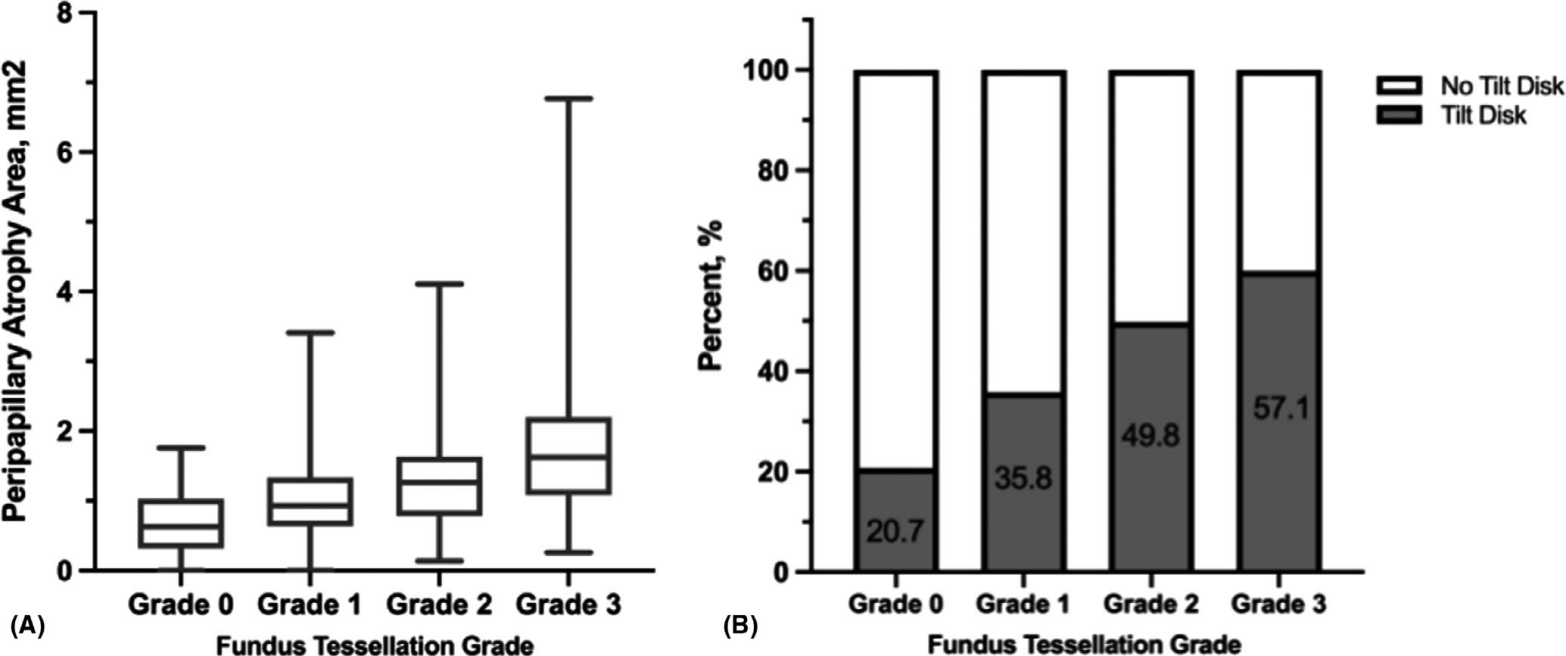
Morphographic changes of the optic disc with increased fundus tessellation.

The optic disc morphological parameters for the different tessellation grades are shown in Table 3. With the development of macular fundus tessellation, the PPA area also became larger (p < 0.001, p‐trend < 0.001), as did the largest PPA diameter (p < 0.001, p‐trend < 0.001). The ovality index showed an increasing tendency with the tessellation grade, and the proportion of tilt discs also gradually increased (20.7%, 35.8%, 49.8% and 59.9%, respectively, grade 0 to grade 3, p < 0.001, Fig. 6) from grade 0 to grade 3. However, no significant difference in the optic disc torsion angle was observed (p = 0.085, p‐trend = 0.949).

**Table 3:** Comparison of morphological characteristics among different fundus tessellation grades.

Variables	Grade 0 (*N* = 29)	Grade 1 (*N* = 95)	Grade 2 (*N* = 233)	Grade 3 (*N* = 156)	Total (*N* = 513)	p*	p‐Trend^†^	Post hoc Test^‡^
Peripapillary Atrophy area, mm^2^	0.71 ± 0.46	1.03 ± 0.61	1.34 ± 0.70	1.81 ± 1.00	1.39 ± 0.84	<0.001	<0.001	Grade0 < Grade1<Grade2 < Grade3
Longest PPA Diameter, mm	0.37 ± 0.22	0.47 ± 0.23	0.60 ± 0.24	0.72 ± 0.26	0.60 ± 0.26	<0.001	<0.001	Grade0 < Grade1<Grade2 < Grade3
Tilt Disc, *N* (%)	6(20.7)	34(35.8)	116(49.8)	89(57.1)	245(47.8)	<0.001	/	Grade0 < Grade1<Grade2 < Grade3
Ovality Index	1.18 ± 0.08	1.24 ± 0.15	1.27 ± 0.16	1.31 ± 0.17	1.27 ± 0.16	<0.001	<0.001	Grade0 < Grade1<Grade3
Optic Disc Torsion, degree	−7.23 ± 12.98	5.24 ± 30.11	−5.08 ± 20.86	−4.37 ± 18.93	−3.52 ± 21.52	0.085	0.949	/

PPA = peripapillary atrophy.

* Significant difference was tested by one‐way ANOVA and chi‐square test.

^†^ p‐trend was acquired by trend test.

^‡^ Post hoc tests among tessellation groups were conducted with SNK method.

### Associated factors of fundus tessellation grade

In addition, we conducted a multivariate ordinal regression analysis to explore the factors associated with the fundus tessellation grade (Table 4). After dropping variables with collinearity, in the optimal multiple regression model (Cox and Snell *R* ^2^ = 0.667, Nagelkerke *R* ^2^ = 0.716, McFadden *R* ^2^ = 0.409), a higher grade of fundus tessellation was independently associated with a thinner macular ChT (every 10 µm thinness, OR = 1.734, 95% CI = 1.621–1.856, p < 0.001), a longer AL (OR = 1.368, 95% CI = 1.105–1.695, p = 0.004), a larger PPA area (OR = 1.391, 95% CI = 1.073–1.802, p = 0.013) and the female sex (OR = 1.605, 95% CI = 1.092–2.359, p = 0.016).

**Table 4:** Multivariate analysis of associated factors for fundus tessellation grades.

Variables*	Estimate	Standardized Estimate	OR	95% CI	p
Macular Choroid Thickness, −10 µm	0.551	2.705	1.734	1.621–1.856	<0.001
Axial Length, mm	0.314	0.341	1.368	1.105–1.695	0.004
Parapapillary Atrophy Area, mm^2^	0.33	0.325	1.391	1.073–1.802	0.013
Ovality Index	1.169	0.197	3.218	0.908–11.408	0.070
Age, year	0.022	0.068	1.022	0.960–1.087	0.496
Gender					
Girls	0.473	0.473	1.605	1.092–2.358	0.016
Boys	Reference				

CI = confidence interval; OR = odds ratio.

* Multivariate analysis was performed by ordinal regression.

## Discussion

To our knowledge, this was the first study to investigate the prevalence of fundus tessellation and its associated factors in children and adolescents with high myopia. We applied a newly proposed grading approach with the assistance of the ETDRS grid for fundus tessellation classification. There was a significant negative correlation between the fundus tessellation grade and ChT in both the macular (*R* = −0.763, p < 0.001) and peripapillary regions (*R* = −0.537, p < 0.001). The proportion of cases with more severe fundus tessellation was greater among patients with an older age, a longer AL and a worse SE. In the multivariate ordinal regression model, a higher grade of fundus tessellation was independently associated with a reduced macular ChT, a longer AL, a larger PPA area and the female sex.

Fundus tessellation (tessellation grade worse than grade 0) in the macular area was observed in 484 (94.3%) eyes, which is a much higher rate than that reported in previous studies. Wong et al reported that the prevalence of fundus tessellation was 54.6% at baseline in highly myopic adolescents with SE worse than or equal to −5 D (12–16 years old; mean age, 14.0 ± 1.0 years; mean SE, −6.2 ± 1.3 D) and increased to 76.1% at the 10‐year follow‐up (mean ± SE, −7.5 ± 1.8 D) (Wong et al. 2018). The prevalence was even lower in a study by Xiao, at 20.0% for the entire population and 12.1% for participants aged 7–18 years under more strict inclusion criteria (SE ≤−6 D) (Xiao et al. 2018). This result could be explained by the following reasons. First, in the above two studies, the International Photographic Classification and Grading System for Myopic Maculopathy (Meta‐PM) was adopted for the classification of myopic maculopathy on colour fundus photography. Through this system, fundus tessellation is defined as well‐defined choroidal vessels that can be observed clearly around the fovea and the arcade vessels (Ohno‐Matsui et al. 2015). While applying this new grading standard, a fundus with any visible choroidal vessels was counted as fundus tessellation, and its level was evaluated by the relative location from the fovea, which may be the major cause of the high prevalence of fundus tessellation. Second, the above two studies included patients with pathological myopia, which were excluded from the final analyses in our study, resulting in the proportion of fundus tessellation being smaller. Finally, based on the Meta‐PM standard, which equals fundus tessellation grade 3 in our study, our prevalence of tessellated fundus among high myopic children and adolescents (30.4%, Shanghai, China) was higher than that in Xiao’s study (12.1%, Guangzhou, China) but lower than that in Wong’s study (54.6%, Singapore). This phenomenon may be due to the age discrepancy, as well as the presence of different social or environmental conditions.

Our findings are in agreement with previous studies showing that fundus tessellation was strongly and negatively correlated with the ChT in both the macular and peripapillary areas, but the differences in the ChT with the tessellation grade were uneven among different regions. In the Beijing Eye Study, the researcher graded tessellation by its visibility using a series of standard photographs and found that higher tessellation grades were associated with thinner subfoveal ChT values in an older population (Yan et al. 2015). In another school‐based study in Beijing, the subfoveal ChT and the ChT 3‐mm nasal and temporal to the fovea were correlated with the tessellation grade among junior high school students when adopting the same grading approach as that used in the Beijing Eye Study (Guo et al. 2019). Yoshihara et al. proposed an objective grading system for fundus tessellation based on transforming the RGB (red, green, blue) pixels of fundus images into three tessellated fundus indices (TFIs) and reported an association between the ChT and these TFIs in the region between the fovea and the optic disc, as well as in the peripapillary region (Yoshihara et al. 2014; Yamashita et al. 2020). However, the ChT in different regions in the macular and peripapillary areas did not decrease evenly with increasing fundus tessellation grade. We found that in the macular area, the subfoveal region and the outer temporal region showed smaller changes in the ChT from grade 0 to grade 1 than any other region; in the peripapillary area, the ChT in the outer temporal region changed the most. This result is consistent with our previous findings, showing that the ChT in the region between the fovea and the optic disc (temporal to the optic disc) decreased the most as myopia worsened, representing the area where the eyeball wall was more likely to be under tension (Deng et al. 2018).

We found a significant association between the tessellation grade and AL, which has also been reported as a risk factor for myopic maculopathy in previous studies. With AL elongation, several fundus layers, such as the choroid and retina, are stretched and thinned; correspondingly, the visibility of large choroidal vessels increases. Thus, in addition to the subfoveal ChT, the AL was another independent factor associated with the tessellation grade in the above two studies in Beijing (Yan et al. 2015; Guo et al. 2019). Furthermore, Xiao et al. reported that a longer AL was associated with more severe myopic maculopathy (OR = 2.97, p < 0.05) in children and adolescents based on the Meta‐PM classification system (Xiao et al. 2018). In Chen’s study, participants with an AL longer than 30 mm had a 6.377‐fold higher risk of having more severe myopic atrophy maculopathy than those with an AL between 26 and 28 mm based on the newly proposed ATN classification system (p < 0.001) (Chen, He, Hu, et al. 2019). Additionally, as longer eyes have been reported to have a higher risk of myopic maculopathy progression (Fang et al. 2018), more highly myopic children should be included in close follow‐up due to the potential for sharp AL elongation during puberty.

We also investigated the relationship between fundus tessellation in the macular area and morphological characteristics of the optic disc. The results indicate that for every 1‐mm^2^ enlargement of the PPA area, there was a 1.391‐fold risk of a higher grade of macular tessellation (OR = 1.391). Similarly, the PPA area was also reported to be a risk factor for fundus tessellation in the Beijing Eye Study, even though different tessellation grading methods were adopted (Yan et al. 2015). Previously, in addition to the tessellation grade, several studies have shown that a larger PPA area is associated with the progression of myopic maculopathy (Fang et al. 2018; Yan et al. 2018). Even among young adults with no myopic maculopathy, eyes with a larger PPA area had a significantly thinner ChT (Chen, He, Yin, et al. 2019). Furthermore, although the optic disc tilt was excluded from the multivariate regression model, it still showed an obvious increasing tendency with increasing fundus tessellation grade. The presence of disc tilt represents stretching of the temporal region of the optic disc, where the ChT varied the most with increasing tessellation grade (Chen, He, Yin, et al. 2019). Therefore, there was a close correlation of tessellated lesions in the macular area with morphological changes of the optic disc.

Finally, females were at a higher risk of having more severe fundus tessellation, which is consistent with the findings of some previous studies. Wei et al. found that the male sex was negatively correlated with the tessellation grade in the Beijing Eye Study (Yan et al. 2015). The female sex has also been reported to increase the risk of myopic maculopathy progression (Fang et al. 2018). Recently, a meta‐analysis revealed that females had a higher risk of myopic macular degeneration than males (Zou et al. 2020). However, its internal mechanism remains unclear.

Overall, the novel grading method for fundus tessellation could be conducive to performing more accurate evaluations of myopic maculopathy and may be more suitable for children and adolescents. The Meta‐PM classification system is primarily based on data from the adult population. As the choroid gradually thins with ageing (Ikuno et al. 2010), a tessellated fundus could be seen in some eyes among the nonmyopic adult population (Yoshihara et al. 2014). The strict diagnostic criteria for fundus tessellation are beneficial for distinguishing those patients with myopic maculopathy. However, fundus tessellation is rarely seen in nonmyopic eyes among children and adolescents, as they were still undergoing a rapid stage of ocular development, during which the ChT would increase normally unless the refraction was worse than −2 D (Xiong et al. 2017). Therefore, for those highly myopic children and adolescents who rarely have any advanced pathological lesions of myopic maculopathy, this hierarchical diagnosis method may help to include more subjects with a potential risk of advanced myopic maculopathy in the monitoring process, especially those without subfoveal tessellation who would not be diagnosed with tessellated fundus with the Meta‐PM standard classification.

In addition, the fast AL elongation may result in significant changes of ChT as well as fundus tessellation, while the tessellation grading seems to be relatively more simple and intuitive than ChT measurement. As fundus imaging equipment is easier to popularize than the SS‐OCT, this grading method of fundus tessellation may be more suitable for primary hospitals. Doctors could monitor the changes in fundus tessellation by observing whether the grade increases. However, the scientificity and feasibility of this tessellation grading approach require further verification.

There were some limitations in this study. First, this novel approach for grading fundus tessellation was only based on colour fundus photographs and involved some subjective bias in critical cases. However, the readers were well‐trained ophthalmologists who were experienced in this field, and a decision was made by another senior ophthalmologist for quality control in the case of disagreement. Second, the number of participants with a low degree of tessellation was relatively small. However, it did not affect the score of the test for the proportional odds assumption in the multivariate ordinal regression model (p > 0.05), and we will try to make comparisons among less myopic children and adolescents in the future. Third, this study followed a cross‐sectional design and could not confirm the exact population at risk for the progression of myopic maculopathy. Longitudinal data are still being collected.

In conclusion, the fundus tessellation grade in the macular area is independently associated with the macular ChT, AL, PPA area and sex of highly myopic children and adolescents. The fundus tessellation grade could represent the degree of choroidal thinning and may be used as a potential index for the assessment of early‐stage myopic maculopathy, especially in young populations. Longitudinal studies are needed to show the changes as tessellation grades increase.
